# Eggshell powder as a biomimetic alternative to biodentine in primary teeth pulpotomy: a comparative clinical and radiographic study

**DOI:** 10.1038/s41598-026-61564-w

**Published:** 2026-07-14

**Authors:** Shaimaa S. El-Desouky, Rehab F. Ghouraba, Ibrahim A. Kabbash, Nancy M. Metwally

**Affiliations:** 1 Pediatric Dentistry, Oral Health, and Preventive Dentistry Department, Tanta University, Tanta, Egypt; 2 Oral Medicine, periodontology, Oral Diagnosis and Radiology Department, Faculty of Dentistry, Tanta University, Tanta, Egypt; 3 Public Health & Community Medicine Department, Faculty of Medicine, Tanta University, Tanta, Egypt

**Keywords:** Pulpotomy, Eggshell powder, Biodentine, MTA, Primary teeth, CBCT, Root resorption, Radiodensity, Sustainable biomaterials, Upcycling., Diseases, Health care, Medical research

## Abstract

Eggshell powder (EP) has been suggested as a cost-effective pulpotomy material due to its high biocompatibility, remineralization potential, and ability to induce dentin bridge formation. This study compared the clinical and radiographic outcomes of EP, Biodentine, and mineral trioxide aggregate (MTA) when used as pulpotomy agents in primary molars. A randomized split-mouth clinical trial was performed on 30 children, each receiving pulpotomy in 3 primary molars assigned to Group I (EP), Group II (Biodentine), and Group III (MTA), with 30 teeth per group. Clinical evaluations were carried out at baseline, 3-, 6-, and 12-month. Cone-beam computed tomography (CBCT) scans were performed immediately postoperatively and at 12- month to evaluate root resorption and radiodensity changes. All groups demonstrated 100% clinical success within 3-month. At 12-month, Group I showed significantly higher rates of spontaneous pain, abnormal mobility, fistula, and internal and external root resorption (*p* < .05). CBCT analysis revealed significantly lower radiodensity values in Group I compared with Groups II and III. Group III (MTA) exhibited the most favorable radiographic outcomes, followed by Group II (Biodentine). EP achieved acceptable short-term results; however, its long-term performance was inferior to MTA and Biodentine.

*Clinical significance* EP represents a low-cost, biocompatible, and environmentally sustainable biomaterial derived from recycled natural waste. Despite its eco-friendly and biomimetic advantages, its long-term clinical and radiographic performance remained inferior to Biodentine and MTA, which continue to be the more reliable pulpotomy materials for primary molars.

*Trial registration* NCT05812053, “ComparativeEvaluation of Eggshell Powder in Primary Teeth Pulpotomy”,https://clinicaltrials.gov/. Registered: 2023/04/13.

## Introduction

Pulpotomy is the most widely used procedure for primary teeth with carious or mechanical pulp exposures, provided no signs of radicular pathology are present^1^. The coronal pulp is removed, the radicular pulp is treated with a medicament, and the tooth is restored to preserve function and maintain arch integrity until exfoliation^2^. Since Sweet^3^ introduced formocresol in 1932, it has become the standard pulpotomy agent due to its high success rates^3^. However, concerns about cytotoxicity, mutagenicity, and declining success with longer follow-up prompted the search for safer alternatives^4^.

The paradigm has since shifted toward materials that are not only biocompatible but also bioinductive, promoting pulp tissue regeneration rather than mere preservation^5^. Mineral trioxide aggregate (MTA), initially developed as a root-end filling material, has shown excellent results in vital pulp therapy^6^. MTA provides superior sealing ability and induces cytokine release, stimulating hard tissue formation^7^. Nonetheless, its drawbacks, such as slow setting time, difficult handling, and high cost, limit its routine use^8^.

Biodentine (Septodont, Saint-Maur-des-Fossés, France), a calcium silicate-based cement, was introduced as a dentine substitute with enhanced mechanical properties, bioactivity, and improved handling^9^. Its refined particle size provides a dense, less permeable structure^10^. Several studies^11,12^ demonstrate that Biodentine performs comparably to MTA in pulp therapy, showing favorable inflammatory and regenerative responses^11,12^. Moreover, it offers excellent sealing ability and has been recommended for pulp capping, pulpotomy, root perforation repair, apexification, and retrograde fillings^12^.

Chicken eggshell powder (EP) has recently gained attention as a natural, calcium-rich biomaterial with potential applications in medicine and dentistry^13^. Eggshells contain approximately 94% calcium carbonate along with calcium phosphate, magnesium, and trace elements such as strontium, fluoride, manganese, zinc, and copper^14^. These components are essential for bone and dental metabolism^15^. Eggshell-derived hydroxyapatite has been shown to be biocompatible, cost-effective, readily available, and suitable for use as a regenerative material^16^. Moreover, EP can occlude enamel surface porosities in caries-like lesions, suggesting remineralizing potential^17^.

Previous investigations of eggshell-derived biomaterials have mainly focused on hard-tissue applications. Clinical studies have reported the successful use of eggshell-derived hydroxyapatite as a bone graft substitute for the healing of maxillary bone defects^16^, while most dental investigations have been limited to in vitro studies evaluating the remineralization potential of EP on enamel caries-like lesions and its ability to enhance the bioactivity of calcium silicate-based materials^13–15^. Although eggshell-derived biomaterials have been investigated in bone regeneration, remineralization, regenerative endodontics, and pulp-capping applications, evidence regarding their clinical performance as pulpotomy medicaments in primary teeth remains scarce^18^. Consequently, a significant knowledge gap exists concerning the reparative potential of EP in vital pulp therapy. Addressing this gap may contribute to the development of a low-cost, sustainable, and biomimetic alternative to currently available pulp therapy materials.

Therefore, this study aimed to compare the clinical and radiographic outcomes of EP, Biodentine, and MTA as pulpotomy medicaments in primary teeth. The null hypothesis was that there would be no significant differences in success rates among the 3 materials.

## Materials and methods

### Study setting and ethical considerations

A randomized, controlled, prospective, double-blind clinical trial was carried out at the Outpatient Clinic of the Pediatric Dentistry Department, Faculty of Dentistry, Tanta University, between April 2023 and July 2025. Cone beam computed tomography examinations were performed at the Oral Medicine, Periodontology, Oral Diagnosis, and Oral Radiology Department of the same faculty. The study was prospectively registered at ClinicalTrials.gov under the identifier NCT05812053 (registered on 13/04/2023). Ethical clearance was obtained from the Research Ethics Committee, Faculty of Dentistry, Tanta University (Approval code: #R-PED-9-20-2), in accordance with the principles of the 1964 Helsinki Declaration and its later revisions.

The study protocol, including the radiographic assessment procedures, was approved by the Research Ethics Committee. CBCT was selected because one of the primary outcome measures was the three-dimensional assessment of internal and external root resorption, as well as quantitative radiodensity changes in the furcation region, which cannot be reliably evaluated using conventional two-dimensional radiography because of anatomical superimposition and the presence of developing permanent successors^19^. CBCT was employed as a research-specific imaging modality to obtain standardized three-dimensional measurements of radiodensity and root resorption that could not be reliably achieved using conventional radiography. To minimize radiation exposure, CBCT examinations were restricted to two time points only (immediately postoperatively and at the 12-month follow-up). Furthermore, the smallest available field of view (8 cm × 5 cm) and a voxel size appropriate for the diagnostic task were used for improving root visibility^20^ and in accordance with the (As Low As Reasonably Achievable) ALARA principle^21^. No additional CBCT scans were obtained beyond those specified in the approved study protocol.

Clinical procedures commenced only after obtaining written informed consent from the children’s parents or legal guardians.

### Eligibility criteria

**Fig. 1:**
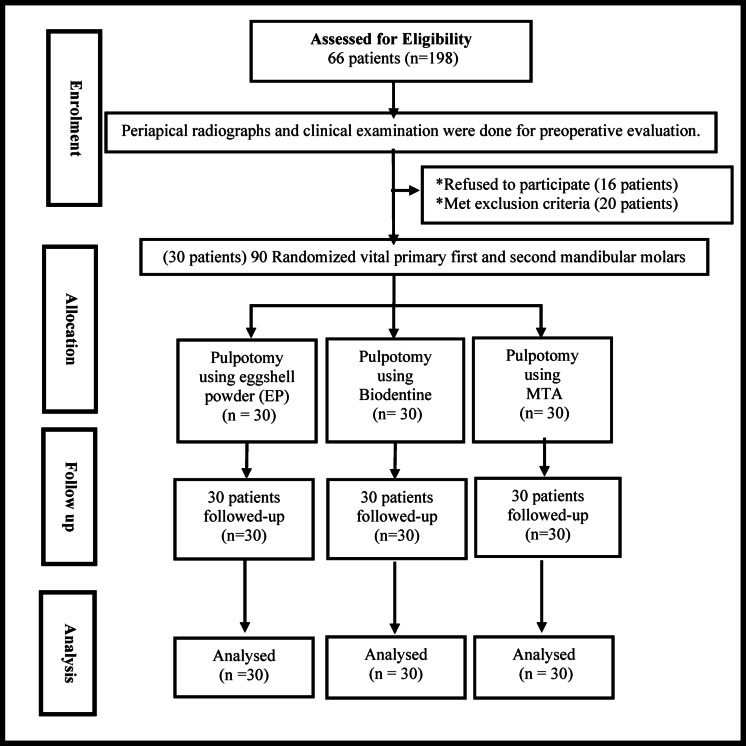
Flowchart explaining the child patient’s randomization and allocation throughout the clinical study.

A total of 198 primary molars from 66 children between 4- and 7-year of age were initially screened according to the study’s inclusion and exclusion criteria. Eligible participants were systemically healthy and cooperative children with restorable primary molars (first or second) affected by deep occlusal and/or proximal caries, categorized as ICDAS code 5 or 6^22^. As confirmed by pre-operative periapical radiographs, selected primary molars were required to have at least two-thirds of the root structure intact^3^ along with normal radiographic findings, including intact periodontal ligament space and absence of internal/external root resorption or interradicular/periapical radiolucency. Exclusion criteria involved primary molars showing irreversible pulpitis signs and symptoms, such as spontaneous pain, tenderness to percussion, sinus tract formation, abnormal mobility, radiographic evidence of inter-radicular pathology, or persistent bleeding exceeding 5 min. Children with systemic illnesses or uncooperative behavior were also excluded. Based on these criteria, 36 children did not qualify for enrollment, resulting in a final sample size of 90 primary molars across 30 children. A CONSORT flow diagram outlining recruitment, allocation, and sample size distribution is provided in Fig. 1. Before intervention, all clinical and radiographic findings were documented during the initial examination.

### Sample size calculation and randomization

Sample size estimation and power analysis were performed using Epi-Info software (version 7.3), developed by the Centers for Disease Control and Prevention (CDC) in collaboration with the World Health Organization (WHO). The calculation was carried out at a 95% confidence interval with 80% study power. Anticipated pulpotomy success rates were assumed to be approximately 58% in the least effective group and 90% in the most effective group. Accordingly, the minimum sample size was set at 30 teeth per group, yielding a total of 90 primary molars for the study.

Random allocation was generated through the Research Randomizer online software (https://www.randomizer.org/). An independent investigator prepared the computer-generated allocation sequence, which was secured in opaque, sealed envelopes to ensure proper concealment until assignment of eligible participants into the three study groups.

### Preparation and sterilization of EP

Using a standardized calcination procedure that was modified from the World Intellectual Property Organization (WO/2004/105912), chicken EP was made^23^. Fresh hen eggs were collected from a local hatchery (Tanta, Egypt). The contents were removed, and the shells were thoroughly washed under distilled water, then boiled at 100 °C for 10 min to facilitate the removal of the inner membrane. Eggshells naturally contain about 94% calcium carbonate (CaCO₃)^24^. Once the shells had dried at room temperature, they were crushed with a sterile mortar and pestle, sieved to create a uniform powder, and then crushed again to get rid of bigger pieces. In order to completely eradicate any pathogens and convert CaCO_3_ into calcium oxide (CaO), the final product was calcined for one hour at 900 °C in a dental laboratory muffle furnace (Kerr Corporation, California, USA)^25^.

CaO, which readily converts to calcium hydroxide [Ca₂]^26^ upon hydration, is produced during high-temperature treatment, thereby promoting alkalinity and imparting bioactive properties beneficial for dental applications^27^. To preserve the physicochemical stability of the prepared powder, moisture uptake and re-carbonation were minimized through controlled cooling and storage in airtight containers. Sterilization was carried out using gamma irradiation, where the gamma cell (Co-60-γ source) operated at a dosage rate of 2 kGy/h. The irradiation procedures were performed at the National Center for Radiation Research and Technology (NCRRT), Atomic Energy Authority (AEA), Nasr City, Egypt, which developed and maintained the irradiation facilities.

### Group assignment

90 primary molars were randomly assigned into three groups using a split-mouth design, with each child having three primary molars: the primary first and second molars on one side of the arch and the primary second molar on the contralateral side.

- *Group-I (experimental group)* (*n* = 30): primary molars were treated with EP mixed with distilled water.
- *Group-II (experimental group)* (*n* = 30): primary molars were treated with Biodentine™ (Septodont, Saint-Maur-des-Fossés, France).
- *Group-III (positive control group)* (*n* = 30): primary molars were treated with MTA (MTA^+^; Cerkamed, Stalowa Wola, Poland).

A split-mouth design was selected to minimize inter-individual variability in factors that may influence pulpotomy outcomes, including age, oral hygiene, dietary habits, caries risk, host response, and occlusal characteristics. By receiving all three interventions within the same patient, each child effectively served as his or her own control, thereby improving comparability among treatment groups and increasing statistical efficiency. Allocation of the pulpotomy materials to eligible teeth was randomized to reduce selection bias and distribute potential anatomical differences among tooth types across the study groups.

### Pulpotomy procedure

All pulpotomy procedures were carried out by the same operator under strict aseptic conditions. Prior to treatment, each case was confirmed clinically and assessed radiographically using a digital periapical photo-stimulated phosphor (PSP) sensor (Apixia HD, Digital Dental Ltd., Westhoughton, Bolton, UK) with standardized exposure parameters of 70 kVp, 6 mA, and 0.8 s. Local anesthesia was administered using 2% mepivacaine with 1:20,000 levonordefrin (Alexandria Co., Egypt), and rubber dam isolation (Midwest Dental, Texas, USA) was applied for all cases.

Caries removal began with a sterile No. 330 carbide bur mounted on a high-speed contra-angle handpiece under continuous water spray. Residual carious dentin was removed with a spoon excavator or a low-speed round bur. Pulp chamber access was gained by removing the chamber roof with a slow-speed round bur, after which the coronal pulp was removed using a large excavator or round bur. The chamber was irrigated with sterile saline, and pulp vitality was assessed; healthy pulp appeared red and vital, with no gray discoloration or signs of necrosis/abscess^28^. Hemostasis was achieved by applying a sterile saline-moistened cotton pellet with light pressure for 3–5 min^29^. If bleeding persisted beyond 5–10 min, the tooth was excluded and treated by pulpectomy^28^.

Following hemostasis, pulpotomy medicaments were applied according to the allocated study group. In group I (*n* = 30), the pulp tissue was dressed with a mixture of EP and distilled water, prepared by combining 1 mg of EP with 5 µL of distilled water to form a slurry that was adjusted to a clinically workable consistency^30^. In group II (*n* = 30), the pulp tissue was capped with Biodentine that was prepared according to the manufacturer’s instructions. The provided liquid was dispensed into the capsule containing the powder and triturated for 30 s at 4,000–4,200 rpm using an amalgamator^31^, producing a homogeneous, clinically workable paste. The material was then placed into the pulp chamber using an amalgam carrier. While in group III (*n* = 30), the pulp tissue was dressed with freshly mixed MTA. The paste was prepared by combining 0.16 g of MTA powder with sterile saline to obtain a uniform consistency^32^. The cavity was subsequently restored with a glass ionomer cement (Medfill, Promedica, Germany), followed by placement of a preformed stainless-steel crown (3 M ESPE, Unitek, United States).

### Clinical and radiographic evaluation

Two qualified blinded pediatric dentists (N.M.M., S.S.E.) conducted the evaluations. Clinical evaluation was conducted at 3-, 6-, and 12-month following the pulpotomy procedures. At every follow-up, the treated teeth were evaluated for clinical signs, including spontaneous pain, tenderness to percussion, abnormal mobility, and the presence of a sinus tract.

Cone-beam computed tomography (CBCT) imaging was performed using a KaVo OP 3D Vision unit (KaVo Dental, Biberach, Germany) immediately after restoration (primary scan) and at the 12-month follow-up (secondary scan). The smallest available field of view (FOV) (8 cm × 5 cm) and a voxel size of 0.125 mm were selected to maximize spatial resolution and reduce patient radiation exposure in compliance with the ALARA principle (*as low as reasonably achievable*)^33^. The imaging protocol employed fixed exposure parameters of 120 kV, 5 mA, 0.125 mm voxel size, and a total exposure time of 7.4 s.

CBCT imaging was not performed as a routine follow-up procedure but rather as a study-specific diagnostic tool to allow standardized three-dimensional assessment of treatment outcomes. The baseline scan served as a reference for subsequent radiodensity measurements and image fusion analysis, while the 12-month scan enabled evaluation of radiographic changes over time in the same anatomical region.

**Fig. 2:**
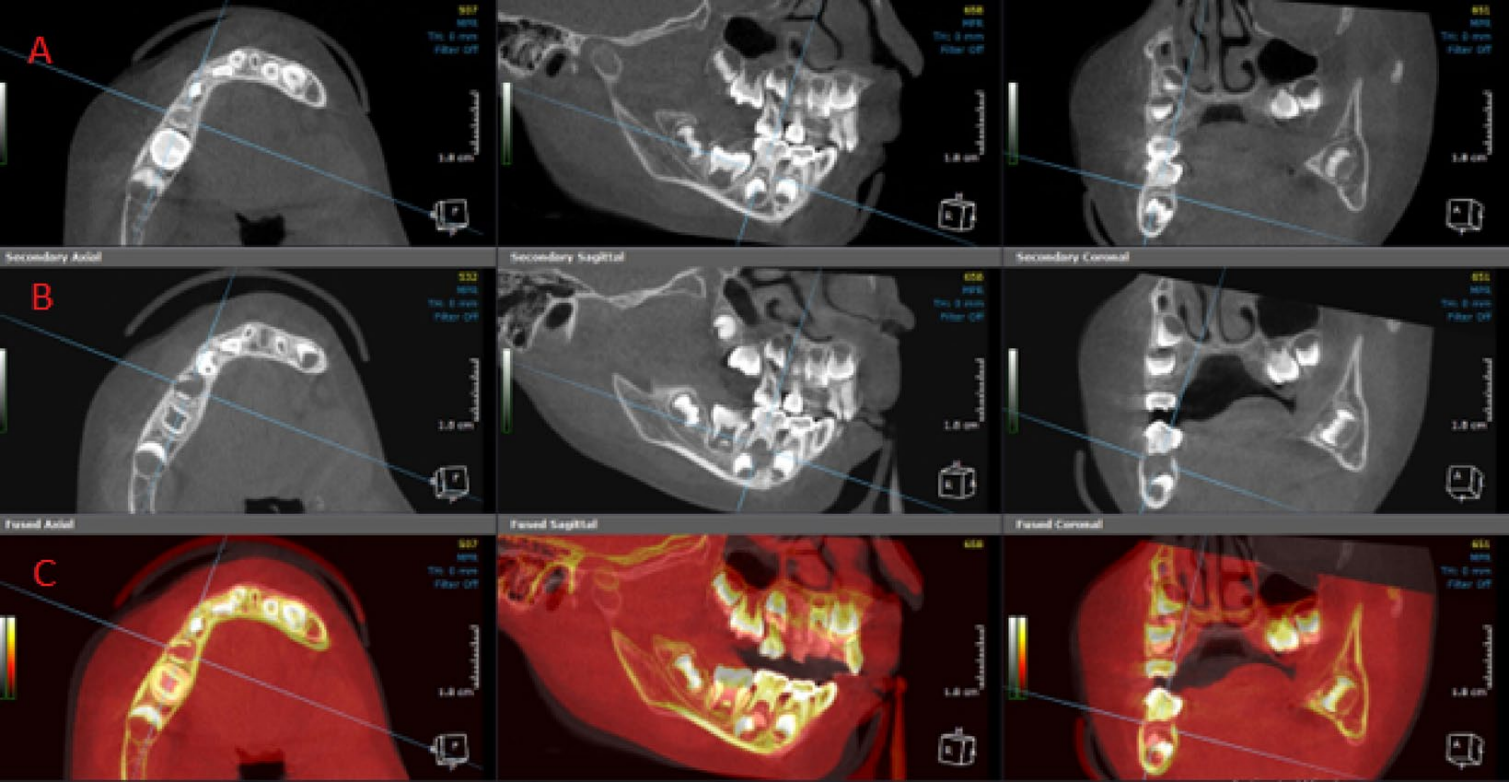
*Fusion On Demand module.* (**A**) Cone-beam computed tomography (CBCT) image obtained immediately after restoration (baseline), (**B**) CBCT image at the 12-month follow-up, and (**C**) superimposed scans demonstrating parallel alignment of the baseline CBCT with the 12-month scan.

The DICOM images from both primary and secondary CBCT scans were analyzed using the OnDemand3D™ software (version 1.0, build 1.0.10.7462, ×64 Edition; Cybermed, 2004–2017; license key 670094709). The fusion module enabled precise alignment of the 2 scans by superimposing one onto the other, thereby eliminating the need for a conventional radiographic paralleling technique. This approach allowed the child to maintain a closed mouth with the chin supported, minimizing discomfort, tension, and fear during image acquisition (Fig. 2).

**Fig. 3:**
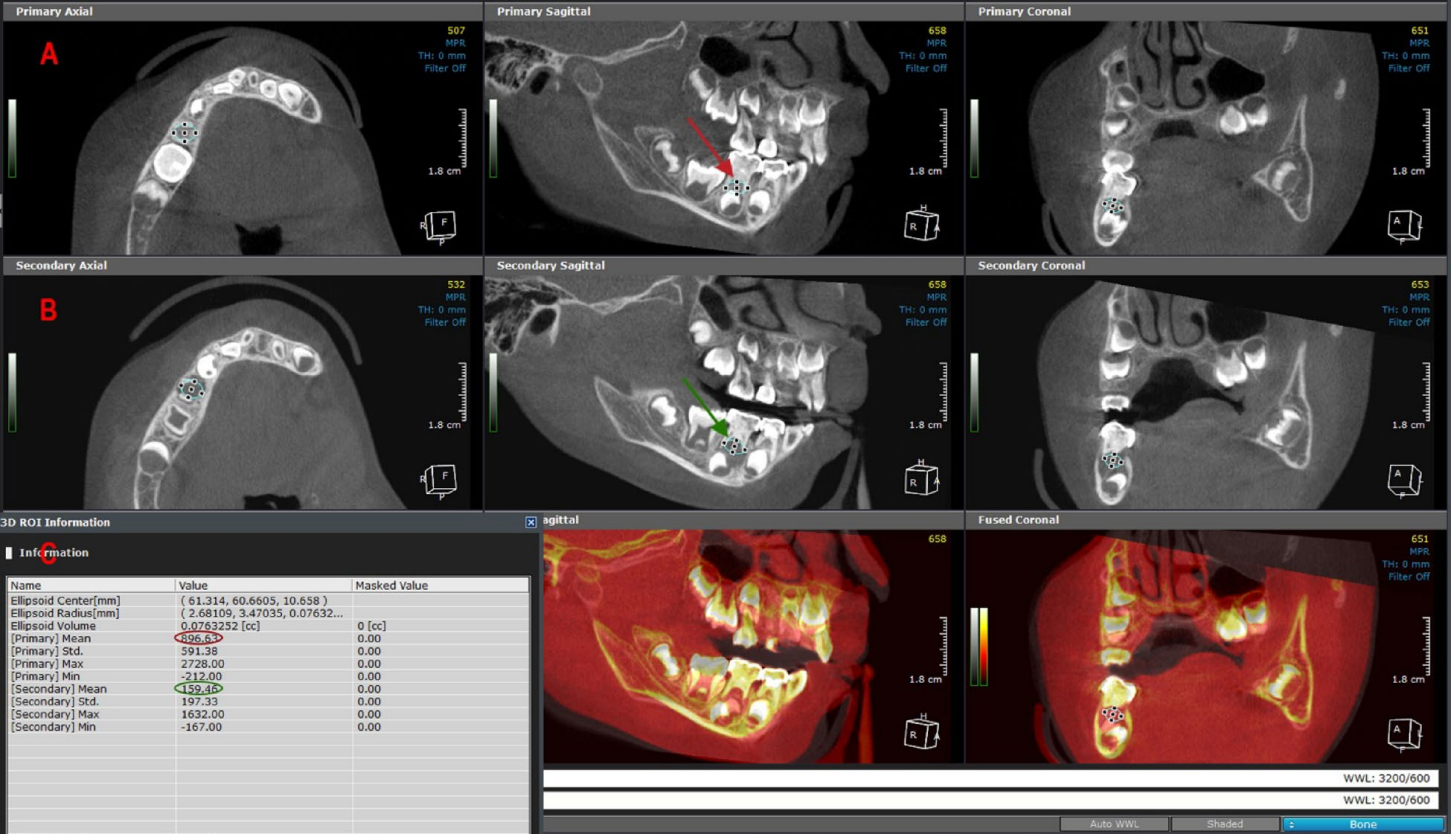
*Region of interest (ROI) in the fusion module of Group I.* (**A**) Density measured at baseline (primary scan) using the ROI tool (red arrow), (**B**) density measured at 12 months post-restoration (secondary scan) using the ROI tool (green arrow), and (**C**) ROI values table showing density at the primary scan (red circle) and at the secondary scan (green circle).

**Fig. 4:**
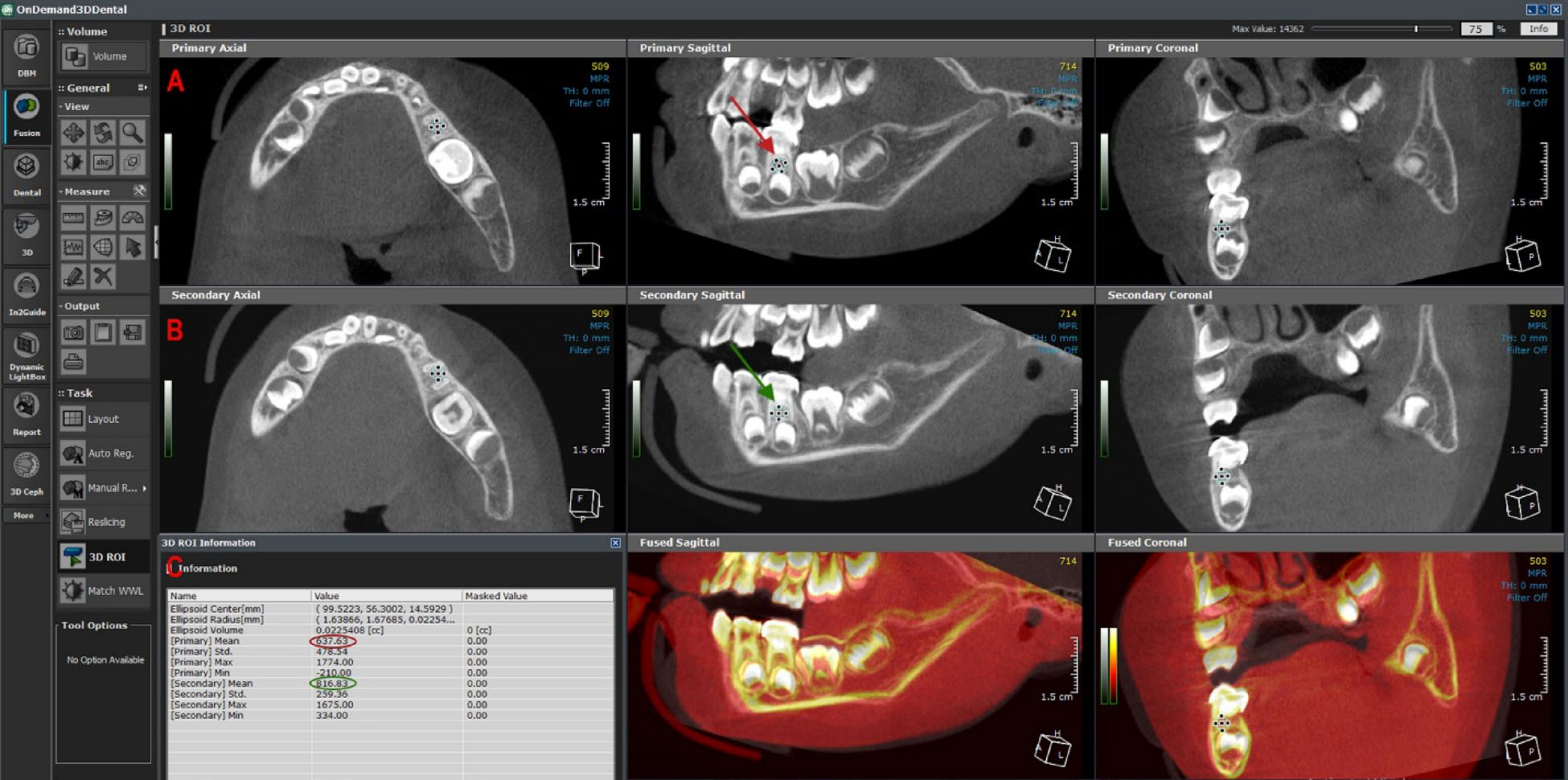
*Region of interest (ROI) in the fusion module of Group II.* (**A**) Density measured at baseline (primary scan) using the ROI tool (red arrow), (**B**) density measured at 12 months post-restoration (secondary scan) using the ROI tool (green arrow), and (**C**) ROI values table showing density at the primary scan (red circle) and at the secondary scan (green circle).

**Fig. 5:**
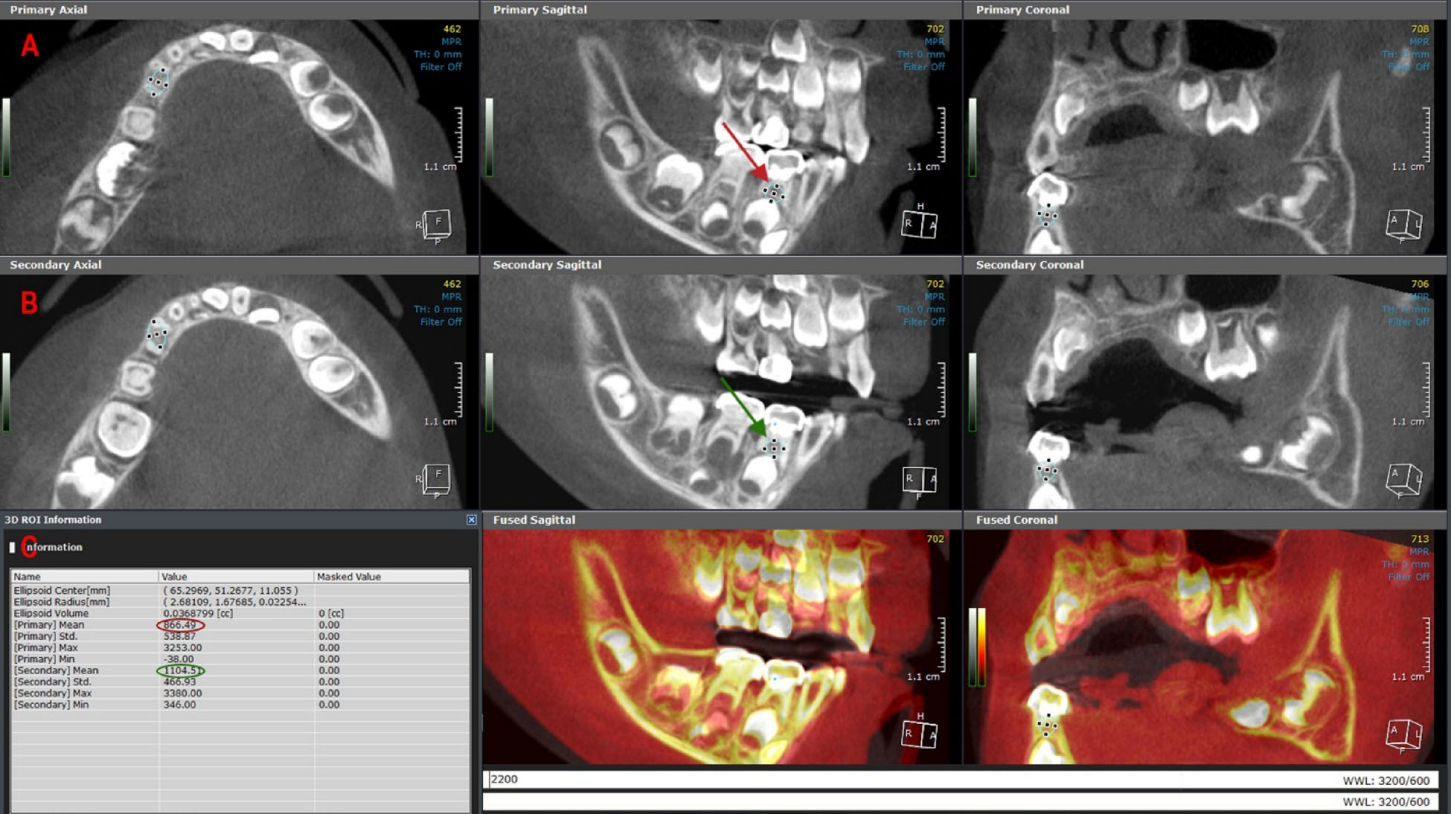
*Region of interest (ROI) in the fusion module of Group III.* (**A**) Density measured at baseline (primary scan) using the ROI tool (red arrow), (**B**) density measured at 12 months post-restoration (secondary scan) using the ROI tool (green arrow), and (**C**) ROI values table showing density at the primary scan (red circle) and at the secondary scan (green circle).

The Region of Interest (ROI) tool within the fusion module was used to simultaneously and synchronously measure radiodensity in the same anatomical location, directly beneath the furcation region, across both scans. Representative ROI measurements are shown in Figs. 3, 4 and 5 for groups-I, II, and III, respectively. Radiographic success was considered when cone beam CT images showed no evidence of internal or external root resorption and no radiolucency in the furcation area.

### Statistical analysis

The collected data were organized, tabulated, and statistically analyzed using SPSS version 26 (Statistical Package for Social Studies), created by IBM, Illinois, Chicago, USA. For radio density, the interquartile range, the mean, standard deviations, and median were calculated. The differences in radio density within each group between baseline and twelve months were tested using the Wilcoxon signed-rank test. The differences in radio density between groups were tested using the Kruskal-Wallis test. For pain, tenderness, mobility, and fistula formation variables, the number and percentage were calculated, and differences between groups were tested by the Monte Carlo exact test. Meanwhile, differences within groups at different follow-up periods were tested using the Friedman test. The level of significance was set at *p*<.05.

## Results

This split-mouth study included 90 primary molars from 30 children, evenly assigned to 3 groups of 30 teeth each. All participants attended clinical and radiographic follow-ups, comprising 43.3% males and 56.7% females. Ages were distributed as 30% aged 4- year, 40% aged 5- year, and 30% aged 6-, 7- year. Tooth type distribution was similar across groups, with first primary molars accounting for 33.3%, 36.7%, and 30.0% in Groups I, II, and III, respectively, and the remainder being second primary molars, with no significant intergroup differences (*p* = .958).

### Clinical evaluation

Regarding spontaneous pain, all patients in the three groups reported no pain at 3- and 6-month follow-up. At the 12- month follow-up, spontaneous pain was reported in 13 cases (43.3%) in the EP group, while no cases of spontaneous pain were observed in the Biodentine or MTA groups, with a statistically significant difference (*p* < .001). Within the EP group, the Friedman test revealed a statistically significant difference in spontaneous pain over time (*p* < .001) (Table 1).

Regarding tenderness on percussion, at 3-month, only the EP group showed 2 cases (6.7%) reporting a positive response to percussion, while Biodentine and MTA remained completely negative (*p* = .333). By 6-month, tenderness in the EP group increased to 4 cases (13.3%), with no cases in groups II or III (*p* = .032). At 12-month, the EP group exhibited a marked increase to 9 cases (30.0%), compared to 2 cases (6.7%) in Biodentine and 1 case (3.3%) in MTA, with a statistically significant intergroup difference (*p* = .007) (Table 1). The Friedman test confirmed a significant increase in tenderness over time in the EP group (*p* < .001), with pairwise analysis revealing significant differences between baseline and both 6- and 12-month (*p* = .046 and 0.003, respectively), as well as between 12-month and both 3- and 6-month (*p* = .008 and 0.023, respectively). At the 12-month follow-up, the EP group differed significantly from both Biodentine (*p* = .021) and MTA (*p* = .006), while no significant difference was found between Biodentine and MTA.

At the 3-month follow-up, all treated teeth across the 3 groups demonstrated complete stability, with no evidence of mobility (100%). By the 6-month evaluation, the EP group exhibited a notable rise in mobility, affecting 4 teeth (13.3%), while Biodentine and MTA continued to show no mobility (*p* = .031). At 12-month, mobility was recorded in 9 teeth (30.0%) in the EP group compared with only one tooth (3.3%) in each of Groups II and III, reflecting a statistically significant intergroup difference (*p* = .003) (Table 1). The Friedman test confirmed a significant time-related increase in mobility within Group I (*p* < .001). Pairwise comparisons in Group I revealed significant differences between baseline and both 6- and 12-month follow-ups (*p* = .046 and 0.003, respectively), as well as between the 3-month and later follow-ups (*p* = .046 and 0.003, respectively).

No fistula formation was observed in any group at 3- or 6-month follow-ups, while at 12- month, 26.7% of teeth in the EP group developed fistula, with no cases occurring in the Biodentine or MTA groups (*p*< .001) (Table 1). The Friedman test showed a significant increase in fistula formation over time within the EP group (*p* < .001).

**Table 1:** Comparative clinical evaluation outcomes of the study groups at successive follow-up periods.

Clinical criteria	Group I (EP) (*n* = 30)		Group II (Biodentine) (*n* = 30)		Group III (MTA) (*n* = 30)		Intergroup *p*-value^†^	
	*n*	%	*n*	%	*n*	%		
*Spontaneous pain*								
Baseline	0	0.0	0	0.0	0	0.0	1.000	
At 3 months	0	0.0	0	0.0	0	0.0	1.000	
At 6 months	0	0.0	0	0.0	0	0.0	1.000	
At 12 months	13	43.3	0	0.0	0	0.0	< 0.001*	
*Friedman test*	39.000		–		–			
*p*	< 0.001*		–		–			
*Pain on percussion*								
Baseline	0	0.0^a^	0	0.0	0	0.0	1.000	
At 3 months	2	6.7	0	0.0	0	0.0	0.333	
At 6 months	4	13.3	0	0.0	0	0.0	0.032*	
At 12 months	9	30.0^bc^	2	6.7	1	3.3	0.007*	
*Friedman test*	18.517		6.000		3.000			
*p*	< 0.001*		0.112		0.392			
*Mobility*								
Baseline	0	0.0^d^	0	0.0	0	0.0	1.000	
At 3 months	0	0.0^e^	0	0.0	0	0.0	1.000	
At 6 months	4	13.3	0	0.0	0	0.0	0.031*	
At 12 months	9	30.0	1	3.3	1	3.3	0.003*	
*Friedman test*	19.909		3.000		3.000			
*p*	< 0.001*		0.392		0.392			
*Fistula formation*								
Baseline	0	0.0	0	0.0	0	0.0	1.000	
At 3 months	0	0.0	0	0.0	0	0.0	1.000	
At 6 months	0	0.0	0	0.0	0	0.0	1.000	
At 12 months	8	26.7	0	0.0	0	0.0	< 0.001*	
*Friedman test*	24.000		–		–			
*p*	< 0.001*		–		–			

*Statistically significant at *p* < .05

† Monte Carlo exact test was used for intergroup comparisons.

Different lowercase superscript letters indicate statistically significant pairwise differences.

### Radiographic evaluation

At the 12-month follow-up, internal resorption was observed in 4 teeth (13.3%) in the EP group, whereas no cases occurred in the Biodentine or MTA groups. Within Group I, the increase from baseline to 12-month was statistically significant (*p* = .046) (Table 2). Similarly, at 12- month, external resorption occurred in 26.7% of teeth in the EP group, compared with 6.7% in Biodentine and 3.3% in MTA (*p*= .017). The Wilcoxon signed-rank test confirmed a statistically significant increase over time in Group I (*p* = .005), while the changes in Groups II and III were not statistically significant (*p* = .157 and 0.317, respectively). Pairwise comparisons at 12-month showed group I significantly differed from both groups II (*p* = .039) and III (*p* = .012), while no difference was found between groups II and III (Table 2).

Regarding the furcation bone radio-density among the 3 groups, at 12-month, radiodensity decreased markedly in group I to 474.2 ± 176.9 HU, while increasing significantly in group II and group III to 936.8 ± 120.8 HU and 1012.1 ± 102.4 HU, respectively (*p* < .001). Within-group changes were significant for all materials (*p* < .001). Pairwise comparisons showed group I differed from groups II and III (*p* < .001), and group II differed from group III (*p* = .014) (Table 3).

**Table 2:** Comparison of internal and external resorption among studied groups at 12-month follow-up period.

Variables	Group I (EP) (*n* = 30)		Group II (Biodentine) (*n* = 30)		Group III (MTA) (*n* = 30)		*p*	
	*n*	%	*n*	%	*n*	%		
*Internal resorption*								
Baseline	0	0.0	0	0.0	0	0.0	1.000	
At 12 months	4	13.3	0	0.0	0	0.0	0.032*	
*Wilcoxon signed-rank test*	2.000		–		–			
*p*	0.046*		–		–			
*External resorption*								
Baseline	0	0.0	0	0.0	0	0.0	1.000	
At 12 months	8	26.7 ^a^	2	6.7	1	3.3	0.017*	
*Wilcoxon signed-rank test*	2.828		1.414		1.000			
*p*	0.005*		0.157		0.317			

*Statistically significant at *p* < .05

Lowercase superscript letter indicates statistically significant pairwise differences.

**Table 3:** Comparison of furcation bone radio-density among studied groups at 12-month follow-up period.

Bone Radio-density	Group I (EP) (*n* = 30)	Group II (Biodentine) (*n* = 30)	Group III (MTA) (*n* = 30)	Kruskall-Wallis test	*p*
Baseline					
Mean *±* SD	729.8 *±* 114.8	740.1 *±* 107.8	779.1 *±* 122.3	3.771	0.152
Inter quartile range	650.7-872.7	667.7-820.2	673.8–877.0		
Median	678.78	734.1	786.06		
At 12 months					
Mean *±* SD	474.2 *±* 1769	936.8 *±* 120.8	1012.1 *±* 102.4	60.473	< 0.001*
Inter quartile range	353.7-564.9	861.2–1000.0	908.7-999.4		
Median	456.78 ^a^	910.39 ^b^	999.44		
*Wilcoxon signed-rank test*	4.618	4.782	4.782		
*p*	< 0.001*	< 0.001*	< 0.001*		

*Statistically significant at *p* < .05.

Different lowercase superscript letters indicate statistically significant pairwise differences.

## Discussion

Pulpotomy remains a critical procedure in pediatric dentistry aimed at preserving the integrity and function of the primary dentition until natural exfoliation. Traditionally, materials such as MTA and Biodentine have demonstrated favorable outcomes due to their bioactivity, sealing ability, and capacity to stimulate dentin bridge formation^34^. However, their high cost and limited availability may restrict widespread use in certain clinical settings^35^. The introduction of EP, a naturally derived calcium-rich biomaterial, presents a novel and economical alternative with promising biological properties, including osteoconductivity, antimicrobial activity, and the capacity to promote tissue regeneration^36,37^. So, this study aimed to explore biocompatible and cost-effective materials for vital pulp therapy in primary teeth. Specifically, it sought to evaluate and compare the clinical and radiographic outcomes of EP, Biodentine, and MTA when used as pulpotomy agents in primary molars.

A major strength of the present study was the use of CBCT for radiographic assessment. Although the paralleling periapical technique is commonly used for follow-up evaluation, obtaining standardized radiographs in pediatric patients can be challenging because of limited cooperation, discomfort associated with film positioning, and anatomical factors that may affect image quality and reproducibility^38,39^. In addition, conventional two-dimensional radiographs may underestimate or obscure root resorption due to the superimposition of surrounding structures, including the developing permanent successor^40^. By contrast, CBCT provides three-dimensional visualization of dental structures, enabling more accurate detection and assessment of treatment outcomes and complications such as internal and external root resorption^41^. Its high spatial approved by selection the smallest resolution allows precise evaluation of root morphology and periradicular structures without the superimposition commonly encountered in conventional radiographs^41^. Furthermore, the use of a limited field of view helped minimize radiation exposure by beam collimation^42^ while maintaining diagnostic accuracy through lowering scatter radiation^43^. These advantages enhance the reliability of the radiographic findings and strengthen the validity of the study outcomes. Also, gaining child co-operation was easier with CBCT than with a periapical radiograph, with chin rest position needing minimal co-operation and the ability of the machine to perform a mock rotation cycle without radiation exposure, familiarizing the patients with the procedure, reducing the child’s anxiety^44^.

The results of the present study partially supported the null hypothesis. No statistically significant differences were observed between Biodentine and MTA in terms of overall clinical and radiographic success across all follow-up periods. However, significant differences were found at the 12-month follow-up regarding spontaneous pain, mobility, fistula formation, internal and external root resorption, and intraradicular bone radiodensity between EP and the other tested materials. Therefore, the null hypothesis was partially rejected.

Clinically, this study demonstrated that all groups achieved 100% success at 3- and 6- months, with no spontaneous pain, tenderness, mobility, or fistula formation. The findings indicated sufficient initial biocompatibility and sealing ability that prevent early postoperative complications such as pain, mobility, or fistula formation^45^. The absence of adverse short-term effects further suggests that EP does not elicit acute inflammatory or cytotoxic reactions, supporting its short-term safety^46^. These findings are consistent with those of Sari et al.,^47^, who demonstrated that eggshell-derived hydroxyapatite exhibited favorable cytocompatibility and no cytotoxic effects on human dental pulp stem cells, with biological behavior comparable to MTA. Furthermore, Aksoy et al.,^48^ reported high calcium and hydroxyl ion release from eggshell-derived hydroxyapatite, indicating considerable bioactivity and the potential to stimulate mineralization and hard tissue formation. Such biological characteristics may explain the excellent short-term clinical outcomes observed in the present study and support the initial healing response of the radicular pulp tissue.

However, by the 12-month follow-up, the EP group showed significantly worse outcomes than Biodentine and MTA. This inferior performance may be attributed to several factors. First, the inferior long-term performance of the EP group may be related to the physicochemical characteristics of the prepared material. Because the EP was subjected to calcination at 900 °C and subsequently hydrated before clinical application, phase transformation from calcium carbonate (CaCO₃) to calcium oxide (CaO) and calcium hydroxide (Ca(OH)₂) likely occurred. However, as no physicochemical characterization analyses were performed, the exact composition of the final material could not be confirmed. Consequently, the observed clinical and radiographic failures may be related to the relatively high solubility and limited long-term dimensional stability of the resulting calcium-based material, which could compromise the pulpal barrier, facilitate microleakage, and permit bacterial ingress over time^49^. In addition, unlike calcium silicate-based materials such as Biodentine and MTA, the prepared eggshell-derived material may not provide the same sustained bioactive reactions and long-term sealing properties required for predictable pulp healing^48^.

Another possible explanation relates to the formulation used in the present study. The EP was mixed with distilled water at a liquid-to-powder ratio adapted from a previously published study^30^ designed for dentin desensitization rather than for vital pulp therapy. This relatively fluid slurry may not possess the same mechanical strength, dimensional stability, or resistance to dissolution as calcium silicate-based materials. The liquid-to-powder ratio used during material preparation may influence porosity, solubility, and mechanical strength. Consequently, a more fluid mixture may exhibit reduced dimensional stability and sealing ability over time, potentially compromising long-term clinical performance^50^. Therefore, the high liquid-to-powder ratio used for EP may have potentially contributed to reduced material stability and sealing effectiveness over time. By contrast, MTA and Biodentine form durable cement-like structures with superior mechanical properties and long-term sealing ability, allowing them to withstand functional stresses and maintain pulpal protection over extended periods^51^.

The present study demonstrated no statistically significant differences between Biodentine and MTA in terms of clinical success throughout the follow-up period. This finding is consistent with previous clinical trials reporting comparable outcomes for both materials when used as pulpotomy medicaments in primary molars^52,53^. The favorable performance of Biodentine and MTA observed in the present study was expected, given their well-documented bioactivity, sealing ability, and long-term clinical success. Consequently, the principal value of the current study lies not in re-establishing the efficacy of these gold-standard materials, but in evaluating the potential of EP as a sustainable, environmentally friendly, and cost-effective biomaterial derived from eggshell bio-waste.

Additionally, at the 12-month follow-up, mobility was detected in one tooth (3.3%) in both the Biodentine and MTA groups. This finding aligns with the observations of Farooq et al.^54^, who reported that primary mandibular first molars are more prone to pulpotomy failure than primary second molars. Therefore, tooth type may have influenced the occurrence of isolated failures in the present study, independent of the pulpotomy material used.

Radiographically, the EP group exhibited significantly higher rates of internal and external root resorption at 12-months, underscoring the superior biocompatibility and pulp-preserving potential of Biodentine and MTA. This outcome may be linked to the intrinsic instability and inferior long-term sealing ability of EP^49^. Internal resorption is typically associated with chronic pulpitis resulting from gradual material degradation, whereas external resorption reflects more severe pulpal inflammation with mediator diffusion into the periodontal ligament via lateral canals and the apical foramen^55^. Both processes arise from persistent low-grade inflammation, likely exacerbated by eggshell powder’s progressive breakdown and microleakage^12^.

The present study results revealed no reported cases with internal resorption at 12-month follow-up in both Biodentine and MTA groups; this was consistent with Holan et al.,^56^ who reported that internal resorption was less frequent in primary molars treated with MTA compared to formocresol following pulpotomy in primary molars. Similarly, Nasrallah and El Noueiri.,^57^ demonstrated 100% radiographic success in primary molars treated with Biodentine, showing no signs of pathological root resorption at 12-month. Also, this was consistent with Cuadros-Fernández et al.^52^, who reported all radiographic failures at 12-month, with success rates of 97% for MTA (1 case of internal resorption) and 95% for Biodentine (1 internal resorption, 1 periradicular radiolucency). Moreover, Bani et al.,^53^ found that the radiographic success rates at 24-month were 93.6% for Biodentine and 87.1% for MTA with no significant differences at all follow-up appointments (*p* > .05). Furthermore, this agreed with the findings of Çelik et al.,^9^ who reported clinical and radiographic success rates at 24-month of 100% for the MTA group and 89.4% for the Biodentine group, with no statistically significant difference (*p* = .646).

The present study demonstrated that while both MTA and Biodentine exhibited substantial increases in radiodensity, EP showed a significant reduction at the 12-month follow-up, indicating inferior hard tissue formation or possible tissue degradation. This finding may be explained by the physicochemical characteristics of the prepared eggshell-derived material. Although eggshell-derived biomaterials exhibit favorable initial bioactivity and calcium ion release, successful long-term performance also depends on material stability, dimensional integrity, and sealing ability^48^. In the present study, progressive material degradation, together with variations in particle size, porosity, and the absence of a standardized formulation, may have contributed to structural instability and the reduction in radiodensity observed over time^49,50^. By contrast, calcium silicate-based materials such as MTA and Biodentine possess superior dimensional stability, sealing ability, and sustained bioactivity, promoting more predictable hard tissue formation and long-term pulp healing^12,58^. This finding aligns with Kim et al.,^59^, who provided micro-CT and histological evidence demonstrating the superior quality of reparative dentin produced by MTA, along with Biodentine’s ability to stimulate mineralization and promote calcified tissue deposition.

The findings of this study suggest that while EP represents a promising, low-cost, sustainable, and naturally derived biomaterial, its current formulation does not provide clinical and radiographic outcomes comparable to those of Biodentine and MTA. The higher incidence of treatment failures and radiographic deterioration observed at the 12-month follow-up indicates that the material, in its present form, lacks the long-term stability required for predictable pulpotomy success. Therefore, unformulated EP cannot presently be recommended for routine clinical use in pediatric vital pulp therapy. Future research should focus on standardizing material preparation, optimizing the formulation, and incorporating suitable stabilizing or bioactive components to enhance its physical properties, sealing ability, and long-term clinical performance.

This study had several limitations. First, although the 12-month follow-up period provided valuable insights, a longer observation period is needed to fully assess long-term clinical and radiographic outcomes, including root resorption and natural exfoliation. The relatively small sample size, while statistically adequate, may limit the generalizability of the findings. Additionally, the inability to blind patients and their parents due to the distinct handling characteristics and appearance of the materials may have introduced bias. Another limitation is that the study included both primary first and second molars. Although treatment allocation was randomized, anatomical and functional differences between these tooth types, including variations in root morphology, pulpal anatomy, physiological root resorption patterns, and exposure to masticatory forces, may have influenced treatment outcomes. Consequently, differences in mobility or radiographic resorption could have been affected not only by the pulpotomy material but also by tooth-specific factors. Future studies may benefit from stratified randomization or separate analyses according to tooth type.

The use of EP, being a novel material with no standardized commercial formulation, also presents concerns regarding consistency and reproducibility. Moreover, no physicochemical characterization analyses, such as X-ray diffraction (XRD), Fourier-transform infrared spectroscopy (FTIR), scanning electron microscopy^20^, or elemental analysis, were performed to verify the chemical composition, phase transformation, particle morphology, or purity of the prepared EP following calcination and sterilization. Consequently, variations in material composition and particle characteristics may have influenced its biological performance. Future studies should incorporate comprehensive material characterization to ensure consistency, reproducibility, and quality control of the prepared biomaterial.

Additionally, the liquid-to-powder ratio used for EP was adopted from a previously published study and was not specifically optimized for pulpotomy applications. The resulting slurry-like consistency may have influenced the material’s mechanical strength, dimensional stability, and long-term sealing ability, potentially affecting the observed clinical and radiographic outcomes.

Although the present study provided valuable clinical and radiographic evidence regarding the performance of EP as a pulpotomy medicament, further long-term clinical studies and histological investigations are required to better elucidate tissue responses and validate these findings.

## Conclusion

Within the limitations of this study, both Biodentine and MTA demonstrated superior clinical and radiographic success in primary molar pulpotomies over a 12-month follow-up period, confirming their reliability as calcium silicate–based biomaterials. Although EP exhibited favorable short-term clinical performance and initial biocompatibility, it failed to maintain comparable long-term outcomes, showing higher rates of clinical failure, root resorption, and lower radiodensity values. These findings suggest that the current formulation of EP lacks the long-term stability and performance required for predictable pulpotomy success. Therefore, unformulated EP cannot presently be recommended for routine clinical use in primary teeth. Further research focusing on material standardization, physicochemical characterization, and formulation optimization is required before its clinical application can be considered.

## Data Availability

The datasets generated and/or analyzed during the present study are available from the corresponding author upon reasonable request.

## Abbreviations

EP: Eggshell powder
MTA: Mineral trioxide aggregate
CBCT: Cone beam computed tomography
FOV: Field of view
HU: Hounsfield units
PDL: Periodontal ligament space
